# Retinal Transcriptomic Signatures in Sudden Acquired Retinal Degeneration Syndrome (SARDS) and Cancer-Associated Retinopathy (CAR)

**DOI:** 10.3390/ani16071051

**Published:** 2026-03-30

**Authors:** Sinisa Grozdanic, Aleksandar Poleksic, Djordje Racic, Dylan Bock, Tatjana Lazic, Markus Kuehn

**Affiliations:** 1 Animal Eye Consultants of Iowa, Hiawatha, IA 52233, USA; dracic@animal-eye-iowa.com (D.R.); tlazic73@gmail.com (T.L.); 2 Department of Computer Science, University of Northern Iowa, Cedar Falls, IA 50614, USA; aleksandarpoleksic@uni.edu (A.P.); bockdaa@uni.edu (D.B.); 3 Department of Ophthalmology, Carver College of Medicine, University of Iowa, Iowa City, IA 52242, USA; markus-kuehn@uiowa.edu

**Keywords:** autoimmunity, SARDS, CAR, retina, molecular

## Abstract

The purpose of this study was to compare the retinal gene expression profiles in canines with Sudden Acquired Retinal Degeneration Syndrome (SARDS) and Cancer-Associated Retinopathy (CAR) and identify shared and distinct molecular pathways. Previously published SARDS and CAR canine retinal microarray data were used for the purposes of retinal transcriptomic pathway analysis, followed by KEGG and GO pathway enrichment analysis using DAVID and MetaCore tools. Gene expression patterns were analyzed to detect the most important signaling pathways. ProteinBERT deep-learning language model, and large language models (LLM-Grok 4, ChatGPT4o) were used for analytical prediction of possible drug targets. Both diseases showed significant upregulation in T-cell co-stimulation and complement activation pathways, including CD86, DLA-79, and C5AR1. Downregulated genes were enriched in pathways associated with visual perception and cardiomyocyte signaling. CAR exhibited upregulation of tumor-related chemokine signaling (e.g., CCR5, CXCR4), while SARDS showed pronounced enrichment in vascular inflammation pathways. Analysis of drug targets identified different classes of drugs, which could be potentially utilized for SARDS and CAR treatment. SARDS and CAR share immune-related molecular signatures but potentially differ in secondary mechanisms—vascular inflammation and endothelial activation in SARDS versus paraneoplastic mimicry in CAR. These data provide potential insight into the pathogenesis of SARDS as well as CAR, and identify potential diagnostic and therapeutic targets.

## Simple Summary

Sudden Acquired Retinal Degeneration Syndrome (SARDS) is one of the most frequent blinding diseases in dogs; however, the cause is not well understood. Cancer Associated Retinopathy (CAR) is a canine blinding disease which shares almost identical clinical presentation with SARDS. This study used computation techniques, deep learning models (DLMs), and large language models (LLMs) to analyze the molecular pathways and predict possible therapeutic targets for treatment of these diseases. Computational analysis of data obtained from retinal tissue of affected dogs revealed strong indication of autoimmune nature of these diseases. Popular Large Language Models (Grok and ChatGPT) were used to generate a possible list of therapeutic targets and medications, which could be used for treatment of these diseases. The generated list of medications was further validated by evaluating it against established machine learning models. This list is, in many aspects, identical to the previously published medications for treatment of autoimmune retinopathies in humans and CAR/SARDS in dogs, validating the hypothesis that SARDS and CAR are likely immune-mediated diseases.

## 1. Introduction

Autoimmune retinopathies (AIRs), such as Cancer-Associated Retinopathy (CAR) and non-paraneoplastic autoimmune retinopathy (npAIR) are characterized by sudden vision loss and progressive photoreceptor dysfunction in humans and dogs [1,2,3]. CAR is considered a paraneoplastic syndrome associated with systemic effects of malignancies and the presence of anti-retinal autoantibodies, while npAIR has not been associated with the presence of cancer [4,5]. Sudden Acquired Retinal Degeneration Syndrome (SARDS) in dogs shares many features of npAIR in humans and has been hypothesized to involve T-cell and complement activation, and autoantibody production, although its etiology is not linked to neoplasia [6,7,8,9,10,11,12,13]. Furthermore, CAR and SARDS in dogs frequently have near identical clinical presentation and response to medical therapy, highly suggestive of very similar etiology.

To better understand the molecular basis of SARDS and CAR, this study employed two computational techniques widely used in transcriptomic data analysis: Kyoto Encyclopedia of Genes and Genomes (KEGG) pathway enrichment analysis and Gene Ontology (GO) enrichment analysis. KEGG categorizes genomic information to broader biological pathways, such as metabolic pathways, regulatory pathways or disease pathways, while GO provides more detailed genomic information by categorizing genes based on their function, cellular location, and biological processes [14,15,16]. KEGG pathway enrichment analysis is a method for identifying functionally grouped sets of genes whose expression is altered in a disease or experimental condition. This analysis evaluates numerous biological pathways for different organs, to reveal whether specific pathways are statistically overrepresented among differentially expressed genes [16]. To date, a direct transcriptomic evaluation of canine SARDS and CAR retinas has not been performed. The objectives of this study were to (1) identify shared and disease-specific molecular pathways in SARDS and CAR retinas using KEGG and GO enrichment analyses on combined and comparative DEG lists, (2) validate key immune pathways at the protein level via immunohistochemistry in available SARDS retinal tissue, (3) quantify serum VEGF levels in a large SARDS cohort, and (4) employ deep-learning language model (ProteinBERT) and large language models (LLMs) Grok and ChatGPT in an exploratory, hypothesis-generating capacity to predict potential therapeutic targets based on the enrichment results.

## 2. Materials and Methods

Previously published SARDS and CAR canine retinal microarray data were used for the purposes of retinal transcriptomic pathway analysis [9,17]. Enrichment analyses were performed using the DAVID 6.8 and MetaCore (version 22.1, https://portal.genego.com/, (accessed on 26 March 2022) Clarivate Analytics, Philadelphia, PE, USA) bioinformatics platforms. Functional categories including KEGG pathways, GO molecular functions, and biological processes were analyzed. Benjamini–Hochberg correction was applied to account for multiple testing and all reported *p*-values were FDR corrected. Visualization of enriched pathways was generated using Seaborn and Matplotlib libraries in Python. The Z-score in MetaCore is a standardized measure of enrichment, based on the hypergeometric distribution (similar to many over-representation analysis tools). It quantifies how far the observed number of hits deviates from what would be expected by chance under a random model. The formula is:

z-score = (actual − expected)/√variance

where: actual = the observed number of objects (genes/proteins) from your dataset that map to the pathway/network; expected = the expected (mean) number of objects in the pathway under the hypergeometric null model, calculated as: expected = (n × R)/N; n = total number of objects in the input list (e.g., differentially expressed genes); R = number of objects in the MetaCore database that belong to the specific pathway/network; N = total number of objects in the MetaCore global network/database background; variance = the variance of the hypergeometric distribution for this setup (typically variance = expected × (1 − R/N) × (N − n)/(N − 1), though exact implementation may use a slight variant for finite population correction). *p*-value is calculated directly from the hypergeometric distribution and ranks results by Z-score or −log(*p*-value). After MetaCore calculates raw *p*-values for enrichment using the hypergeometric distribution, it applies the Benjamini–Hochberg false discovery rate correction procedure to adjust these *p*-values across all tested ontologies or categories, producing FDR-corrected *p*-values. The BH procedure in MetaCore follows the standard step-up method: a) Sort the raw *p*-values in ascending order: p_{(1)} ≤ p_{(2)} ≤ … ≤ p_{(m)}, where m is the total number of tests (e.g., number of pathways or GO terms tested). b) For each ranked *p*-value p_{(k)}, compute the adjusted value as: adjusted_p_{(k)} = min(m × p_{(k)}/k, 1) (with monotonicity enforcement: if adjusted_p_{(k)} > adjusted_p_{(k + 1)}, set adjusted_p_{(k)} = adjusted_p_{(k + 1)}). The largest k where adjusted_p_{(k)} ≤ 0.05 (FDR threshold) identifies the cutoff—all *p*-values up to that rank are considered significant at FDR ≤ 0.05.

ProteinBERT deep learning language model analysis.

All results were generated using ProteinBERT [18], a deep-learning language model built on the BERT architecture. ProteinBERT is a novel deep-learning language method specifically engineered to extract both local and global representations from protein sequences. The method has proven high accuracy in predicting rare protein functions. ProteinBERT’s foundational knowledge stems from pretraining on 106 million proteins sourced from the UniRef90 database, encompassing diverse organisms, including canines. This training integrates Gene Ontology (GO) annotations, which categorize proteins by their biological functions and specific cellular processes.

Model optimization procedure

The pretrained model was fine-tuned in-house to recognize drug-target interactions. We developed three distinct model variants based on three specific datasets:

(A) DrugBank: 1408 proteins and 1482 drugs [19]
(B) BioSnap: 2321 proteins and 5017 drugs [20]
(C) Therapeutic Target Database (TTD): 2669 proteins and 29,993 drugs [21]

The finetuning process followed a structured three-step pipeline. First, the model loaded its pretrained weights to anchor its understanding of protein-GO relationships. Second, the model was trained to map patterns between amino acid sequences and drug interactions using known GO data; this phase typically reached convergence within 15–20 epochs. Finally, the entire pipeline was finetuned to allow the model to update its core protein representations to more accurately reflect the learned drug-target dynamics. This final stage generally concluded within 8–10 epochs. To mitigate overfitting, we implemented an early stopping protocol within a maximum window of 40 epochs, ensuring training termination if accuracy gains reached a plateau. The model output scores were calculated on the scale from 0 to 1, with the average score of ~0.001. More precisely, the *p*-value is calculated as:

(r+1)/(n+1)

where r is the number of scores greater than s and n is the number of samples (100,000).

Large Language Models (LLMs) analysis.

Large language models, GROK 4 (xAI), and ChatGPT 4o (OpenAI) were used for identification of possible drug targets based on the analysis of the generated KEGG and GO data.

Immunohistochemistry (IHC) analysis on canine retinal tissue was performed as previously reported [9]. Briefly, tissue samples for IHC were fixed in 4% paraformaldehyde, embedded in paraffin, and sectioned into 5–7 μm thick sections. Sections were deparaffinized with heat and xylene, and rehydrated by serial rinses in decreasing concentrations of ethanol. Endogenous peroxidase activity was quenched by incubation with 3% H_2_O_2_ for 10 min. Following rinses in potassium phosphate-buffered saline (KPBS), cells were incubated in blocking solution containing 5% normal donkey serum (NDS, 017–000–121; Jackson ImmunoResearch, West Grove, PA, USA), 0.1% BSA (BSA, A9647; Sigma, St. Louis, MO, USA), and 0.04% Triton X-100 for 2 h to eliminate non-specific antibody labeling. Tissue was then incubated in primary polyclonal antibodies overnight at room temperature including: anti-CD3 (T-lymphocyte marker; Dako, Carpinteria, CA, USA); anti-CD79 (B-lymphocyte marker; Dako); and anti-C1q and anti-C4 (complement components C1q and C4; Bethyl Laboratories Inc, Montgomery, TX, USA). Sections were then incubated with a biotinylated secondary antibody (10 min), and this complex was labeled with streptavidin–horseradish peroxidase conjugate and identified with diaminobenzidine, followed by Mayer’s hematoxylin counterstain. Stained tissue sections images were taken with an Axioplan 2 microscope (Carl Zeiss MicroImaging, Inc., Thornwood, NY, USA), equipped with a color camera (AxioCam MRc; Carl Zeiss Meditec USA, Inc., Dublin, CA, USA).

Complement components (C1q and C4) quantification was performed as previously reported [22]. Four images of the central and peripheral retina were taken for each section containing optic nerve (2–4 sections were quantified with data averaging from all sections for each of selected central and peripheral field) using a Nikon Microphot Microscope (Nikon Inc. Garden City, NY, USA) and a 40× oil immersion objective from eyes of 7 control animals, 6 SARDS patients and 1 CAR patient. Central retinal images were obtained within two microscope fields of the optic nerve. Peripheral retinal images were obtained 7–8 microscope fields away from the optic nerve. The microscope settings for tissue stained with a particular antibody were left consistent to eliminate variation from one sample to the next. A blank image that did not contain any tissue was obtained to correct for any slight variations in the background signal noise. Metamorph image analysis software (Ver. 7; Molecular Devices, Sunnyvale CA, USA) was used to quantify the percentage of the retina that was immunoreactive for each antibody. Blank images from the data set were used to correct each slide to account for differences in light illumination. A threshold of two standard deviations below the median staining intensity for each group stained with an antibody was determined, the immunoreactivity was pseudocolored and the fraction of the retina labeled was calculated using Metamorph in the masked manner, so identification information of control and disease-affected sections were not available to evaluator. The immunoreactivity of all retinal layers combined was quantified. Morphometric data were statistically analyzed using Students *t*-test (SARDS vs. control) and Paired *t*-test (SARDS peripheral retina vs. SARDS Central retina expression) on log-transformed data evaluated with Shapiro–Wilk test for normality of distribution using Graphpad Prism (ver. 10; Graphpad Software, La Jolla, CA, USA).

Vascular Endothelial Growth Factor (VEGF) quantitative sandwich enzyme-linked immunoassay (ELISA). Canine serum samples from 59 SARDS dogs, 5 CAR dogs, and 19 healthy control dogs were used for quantification of serum VEGF levels. For quantitative determination of canine vascular endothelial growth factor (VEGF) in canine serum samples, a commercial ELISA kit for canine VEGF_163_ was used (Quantikine^®^ Canine VEGF Immunoassay, R&D Systems Inc., Minneapolis, MN, USA). Briefly, 120 canine serum samples stored at −80 °C were thawed slowly on ice, vortexed gently for 15 s, and 200 μL of each were assayed exactly according to the procedural conditions specified for serum samples by the kit manufacturer’s instructions. Eight serial 1:2-diluted VEGF_163_ standards ranging from 0 to 2500 pg/mL were prepared using the purified recombinant canine VEGF_163_ lyophilate and the RD6U diluent provided in the kit according to the manufacturer’s instructions. All samples and standards were tested in duplicate in the 96-well microplates provided in a single run to avoid variability in testing conditions. Quantification of the reaction was measured using a SpectraMax^®^ 190 UV-Vis Microplate Spectrophotometer (Molecular Devices, San Jose, CA, USA). Results were interpreted using both a 4-parameter logistic curve-fitting model (generated by SOFTmax PRO version 3.1.2, Molecular Devices) and a simple linear model plotting the standard curve as the mean corrected optical density versus pg VEGF/mL (using Microsoft Excel). Bradford protein assays were run for 68 samples to rule out the influence of the protein concentration on VEGF levels, but no correlation or relationship was found between total serum protein and presence of VEGF (*p* = 2.98 × 10^−11^; r^2^ = 0.000758).

## 3. Results

### 3.1. Transcription Regulation Workflow Analysis

**Figure 1:**
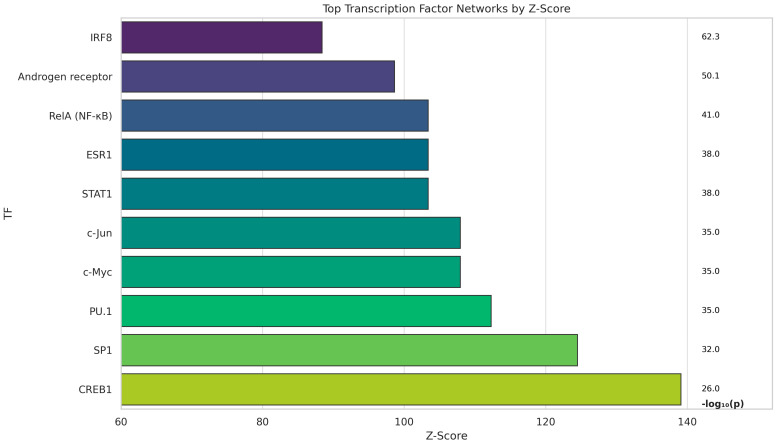
Top shared transcription factor networks in SARDS and CAR retinas. Transcription factor (TF) network enrichment analysis identified the most significantly upregulated TF and associated regulatory networks in combined SARDS and CAR retinal datasets. Each network is ranked by z-score and associated GO biological processes. Key TFs include CREB1, SP1, PU.1, c-Jun, c-Myc, NF-κB (RelA), ESR1, STAT1, androgen receptor, and IRF8. Immune-mediated processes, leukocyte chemotaxis, cytokine signaling, and antigen presentation were dominant processes in SARDS and CAR retinas.

The analysis identified transcription factor-centered networks that are enriched in the input gene lists derived from CAR and SARDS datasets, when compared to healthy canine retinas (Figure 1). Each network was centered on a transcription factor (TF) and included genes regulated by that TF, with associated Gene Ontology (GO) biological processes, statistical enrichment (*p*-value), and scoring (z-score). All of the top TF networks had high statistical significance (z-score > 60), indicating robust enrichment. The most significant transcription factors and their associated biological processes were identified and represented in Figure 1. CREB1 was identified as a top ranked network (Score: *p* = 5 × 10^−63^, z-score = 139.08) for which its respective GO processes identified leukocyte chemotaxis, TNF cytokine production, and defense response as the most important features for this network. Implication of CREB1 network is pertinent to strong immune-mediated processes. CREB 1 was followed by SP1 for which GO processes identified response to external stimuli and biotic factors as main features. SP1 is a broad regulator of inflammatory and stress responses. PU.1 (SPI1) network suggests enhanced antigen presentation and macrophage/dendritic cell activity (GO processes: innate and adaptive immune responses). Following was a group of TFs c-Jun, c-Myc, and NF-κB (RelA) which are associated with cellular stress, chemotaxis, and immune regulation. These TFs are classical mediators of inflammation and apoptosis; upregulated networks may indicate tissue injury response and possibly immune checkpoint dysregulation. ESR1 (Estrogen Receptor 1) is TF which modulates immune cell development, function of immune cells and inflammatory responses. STAT1 enhances inflammation, and innate and adaptive immunity. Androgen receptor is expressed in various immune cells (neutrophils, macrophages, B-cells and T-cells) and generally has immunosuppressive effects. The IRF8 TF plays important role in development and function of dendritic cells, monocytes, and macrophages.

### 3.2. GO Enrichment Analysis

**Figure 2:**
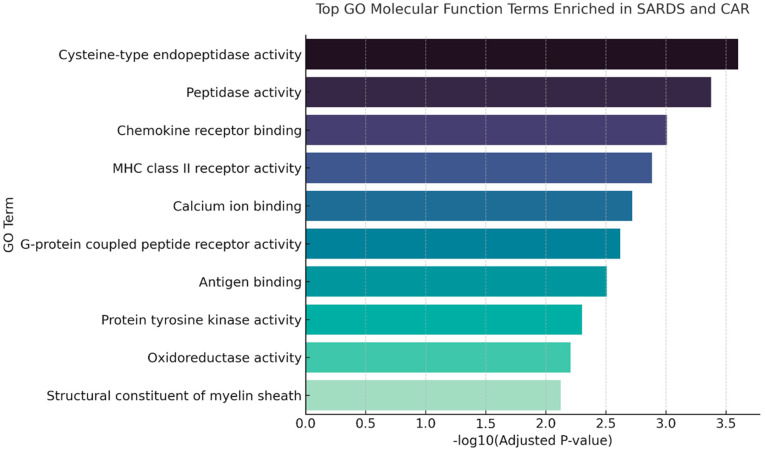
Shared Gene Ontology (GO) molecular function enrichment from SARDS and CAR retinas. Top GO molecular function categories significantly enriched in the combined SARDS and CAR dataset. The most prominent terms include cysteine-type endopeptidase activity, peptidase activity, chemokine receptor binding, MHC class II receptor activity, protein tyrosine kinase activity, and oxidoreductase activity.

Gene Ontology (GO) molecular function enrichment analysis identified several functional categories significantly overrepresented in the combined SARDS and CAR dataset when compared to healthy controls (Figure 2). The most strongly enriched terms included cysteine-type endopeptidase activity (CASP4, CTSS) and peptidase activity (LOC479458, CTSS) indicating involvement of proteolytic processes, likely as a result of apoptosis/cellular death, but these genes are also involved in processing antigens for presentation by the Major Histocompatibility Complex (MHC). Additional highly enriched categories included chemokine receptor binding and MHC class II receptor activity (CCR5, CXCR4, C5AR1), further suggesting immune-related signaling and antigen presentation components. GO terms, such as calcium ion binding, G-protein coupled peptide receptor activity, and antigen binding, further emphasize the importance of immune-mediated signaling. Enrichment of protein tyrosine kinase activity and oxidoreductase activity points to alterations in intracellular signaling and redox processes. Structural constituent of myelin sheath was also significantly enriched, which may reflect changes associated with neuronal death and glial structural responses.

### 3.3. KEGG Pathway Enrichment Analysis from SARDS and CAR Retinas

**Figure 3:**
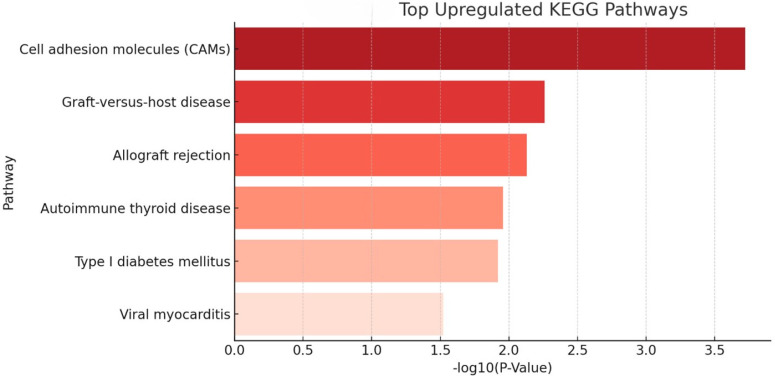
KEGG pathway enrichment—shared upregulated pathways from SARDS and CAR retinas. The most enriched pathway is cell adhesion molecules (CAMs), followed by immune-related pathways including graft-versus-host disease, allograft rejection, autoimmune thyroid disease, type I diabetes mellitus, and viral myocarditis. Observed changes are potentially indicative of shared T-cell co-stimulation and antigen presentation mechanisms dominating pathways in SARDS and CAR retinas.

KEGG pathway enrichment analysis revealed distinct functional patterns for upregulated and downregulated genes in SARDS and CAR relative to controls. Among the upregulated pathways, the most significantly enriched was cell adhesion molecules (CAMs) pathway, followed by several immune-related pathways including graft-versus-host disease, allograft rejection, autoimmune thyroid disease, type I diabetes mellitus, and viral myocarditis (Table 1, Figure 3). These results indicate a strong immune activation and antigen presentation signature in both diseases.

**Figure 4:**
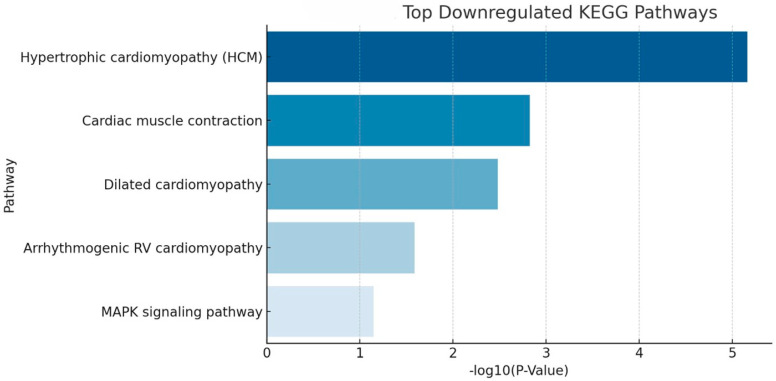
KEGG pathway enrichment—shared downregulated pathways in SARDS and CAR retinas. The most significantly suppressed pathways include hypertrophic cardiomyopathy (HCM), cardiac muscle contraction, and visual perception. Additional downregulated pathways are observed in MAPK signaling.

**Figure 5:**
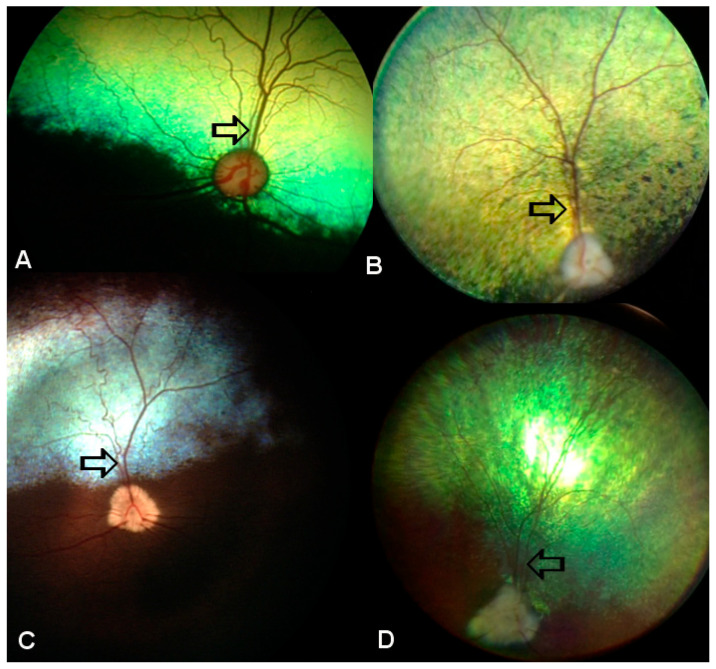
Fundus image of SARDS patients reveals vascular attenuation and thinning of retinal veins and arterioles. (**A**) Control healthy canine retina; (**B**–**D**) fundus image from SARDS patients. Black arrow points to the dorsal retinal vein showing thinning in SARDS retinas.

Downregulated pathways were predominantly associated with cardiac and contractile functions, with the most significant suppression observed in hypertrophic cardiomyopathy (HCM). Additional significantly downregulated pathways included cardiac muscle contraction, dilated cardiomyopathy, and arrhythmogenic right ventricular cardiomyopathy, along with a signaling-related reduction in the MAPK signaling and visual perception pathways (Table 2, Figure 4 and Figure 5). The observed pattern suggests that while immune activation is markedly increased, certain structural and signaling pathways related to vision and retinal vasculature are concurrently decreased. These findings are congruent with our previously published clinical observations [9,17] and confirmed here by the retinal vascular attenuation in clinical patients observable in patients (Figure 5).

**Figure 6:**
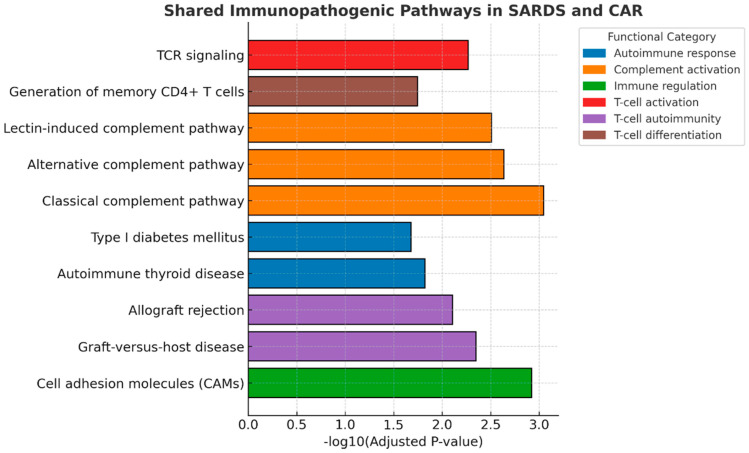
Shared immune-focused KEGG pathway analysis in SARDS and CAR retinas. Immune-related KEGG pathway mapping revealed upregulation of T-cell activation, complement activation, and leukocyte adhesion pathways in SARDS and CAR retina, potentially implying the significant role of adaptive and innate immune activation in disease pathogenesis.

More focused analysis of KEGG pathways toward immune-mediated process networks, revealed that T-cell activation and complement activation pathways may potentially play a significant role in the SARDS and CAR molecular mechanisms responsible for retinal damage (Figure 6).

### 3.4. Comparative Analysis of SARDS and CAR

**Figure 7:**
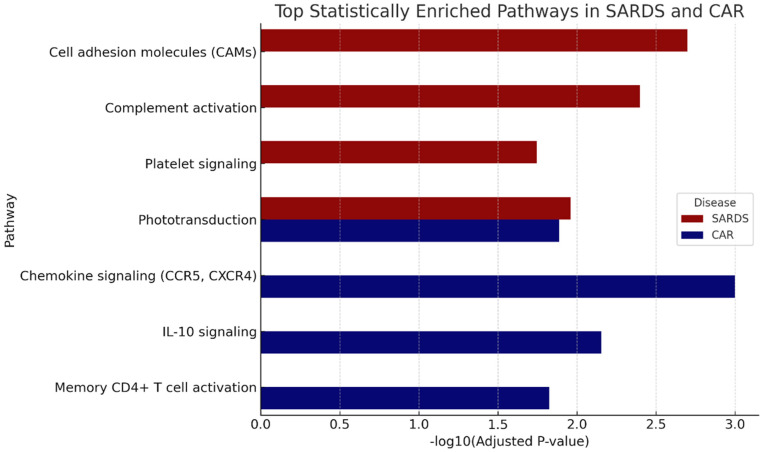
Differential immune pathway expression between SARDS and CAR. Differential KEGG pathway enrichment between SARDS and CAR. CAR demonstrated stronger expression of chemokine signaling genes (e.g., CCR5, CXCR4), potentially indicative of the paraneoplastic immune modulation in CAR retinas. SARDS showed greater enrichment for complement and coagulation pathways (e.g., C5AR1), potentially indicative of vascular inflammation and innate immune activation.

Although SARDS and CAR share many transcriptomic features, notable differences in gene expression patterns were observed (Figure 7). Both conditions showed significant enrichment in immune-related KEGG pathways such as cell adhesion molecules and antigen presentation. However, CAR samples displayed relatively higher expression of cancer-related immune modulation genes (e.g., CCR5, CXCR4), consistent with paraneoplastic immunological pathways. In contrast, SARDS samples showed stronger enrichment of complement and coagulation-related genes (e.g., C5AR1, CD86).

Analysis of genes expressed at lower levels indicated that both conditions exhibited reduced abundance of genes involved in phototransduction (e.g., PDE6A, CNGA1) and cardiac muscle contraction (e.g., CACNB2, TNNI3). The decrease in photoreceptor associated transcripts is most likely due to the pronounced loss of photoreceptor cells which is the hallmark of both conditions. The magnitude of downregulation in cardiac-related pathways was more pronounced in SARDS, most likely as a result of the cytoskeleton changes due to retinal neuronal damage and loss, but could also potentially reflect systemic and retinal vascular comorbidities.

### 3.5. Immunohistochemistry Conformation of Immune Cells in SARDS Retinas

**Figure 8:**
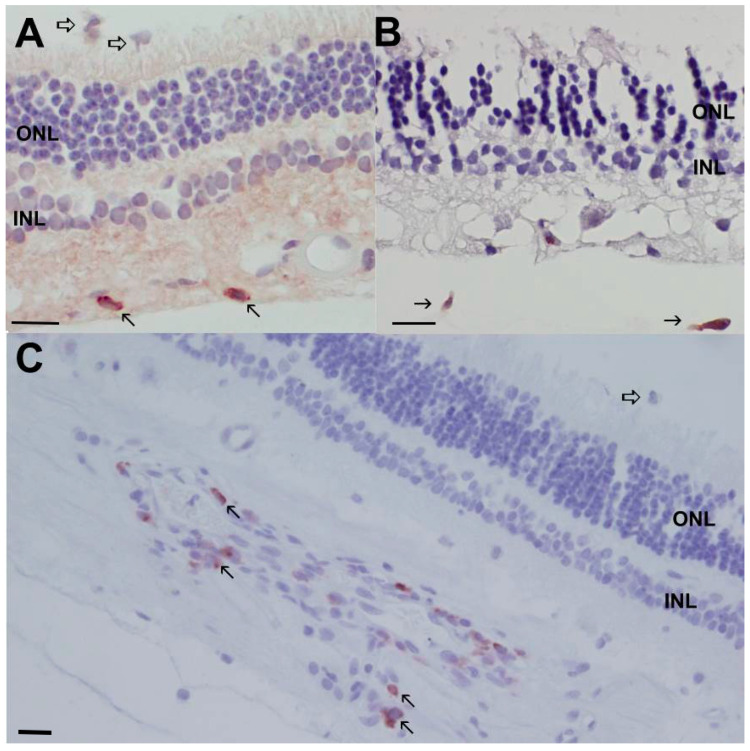
Immunostaining with anti- T and anti-B-cell antibodies of canine SARDS retina. (**A**) Immunostainings of canine SARDS retina with anti-CD3 antibodies (T-cell marker). Micrograph shows loss of outer segments and infiltration of the outer segment layer with inflammatory cells (open arrow). Sporadic presence of T-cells (CD3 positive cells marked by closed arrow) is evident in the nerve fiber layer. (**B**) Retinal immunohistochemistry analysis with the use of plasma cell marker (Ig cocktail of antibodies was used) shows sporadic presence of Ig positive cells (closed arrows) in the anterior vitreal surface. (**C**) Immunohistochemistry analysis of canine SARDS retina with the use of B-cell marker (CD79). Micrograph shows loss of outer segments and infiltration of the outer segment layer with inflammatory cells (open arrow). Perivascular presence of B-cells (CD79 positive cells marked by closed arrow) is evident in the nerve fiber layer. All patients presented with a sudden onset of complete blindness and completely extinguished retinal electrical activity. INL—inner nuclear layer; ONL—outer nuclear layer; bar = 15 μm.

In order to evaluate retinas for the presence of immune cells, immunohistochemistry analysis for T-cell marker (CD3) and B-cell marker (CD79) was performed in canine SARDS and control retinas. Immunohistochemistry analysis showed the presence of T- and B-cells in SARDS retinas, particularly in the perivascular regions (Figure 8), but not in the control retinas.

### 3.6. Immunohistochemistry Confirmation of Complement Activity in SARDS Retinas

**Figure 9:**
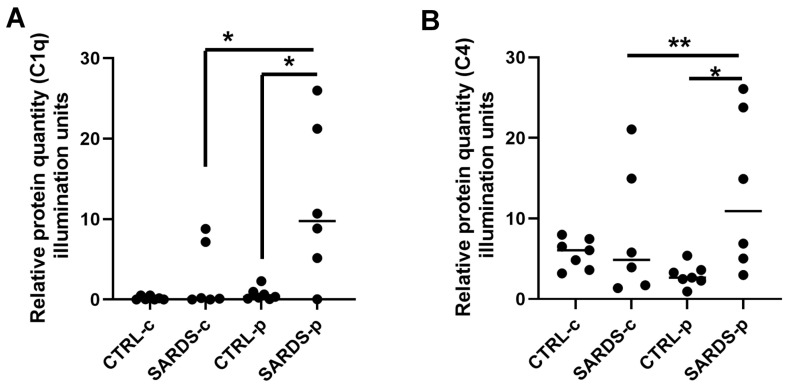
Quantification of complement component protein expression (C1q and C4) in SARDS retinas. Quantification of immunohistochemistry reactivity showed statistically significantly increased protein expression in the peripheral retinal region of SARDS dogs for C1q (**A**) and C4 (**B**) complement components. SARDS-c- SARDS central retina regions, SARDS-p—SARDS peripheral retinal regions, CTRL-c—control healthy dog central retinal regions, CTRL-p—control healthy dog peripheral retinal regions * *p* < 0.05; ** *p* < 0.001.

**Figure 10:**
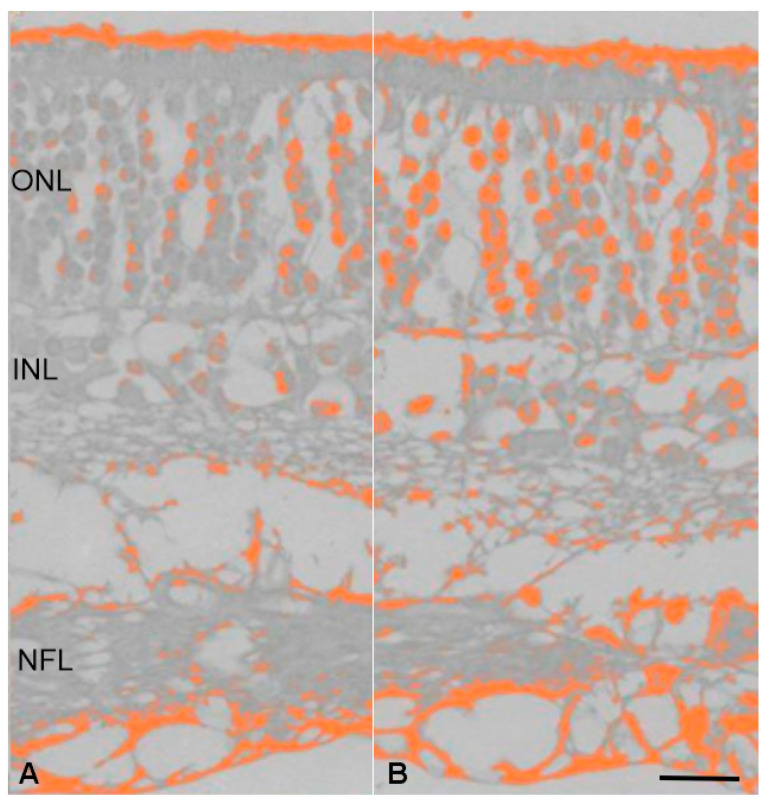
Quantification of complement component 4 (C4) protein expression in canine CAR retinas. Pseudocoloring of immunohistochemistry reactivity showed increased complement component deposition. (**A**) Pseudocoloring of the complement deposits in the control retina; (**B**) pseudocoloring of the complement deposists in CAR retina. NFL-nerve fiber layer; INL-inner nuclear layer; ONL-outer nuclear layer; bar = 15 μm.

In order to confirm complement activity, immunohistochemistry evaluation and quantification of the expression signal was performed which revealed significantly increased deposition of complement components expression in SARDS retinas when compared to the healthy control retinas. Furthermore, our analysis revealed increased complement accumulation in the peripheral retinal regions of SARDS dogs when compared to the central retinal regions of SARDS dogs (Figure 9). Similar changes have been observed in CAR canine retinas (Figure 10).

### 3.7. Analysis of Vascular Endothelial Growth Factor (VEGF) in Canine Serum

**Figure 11:**
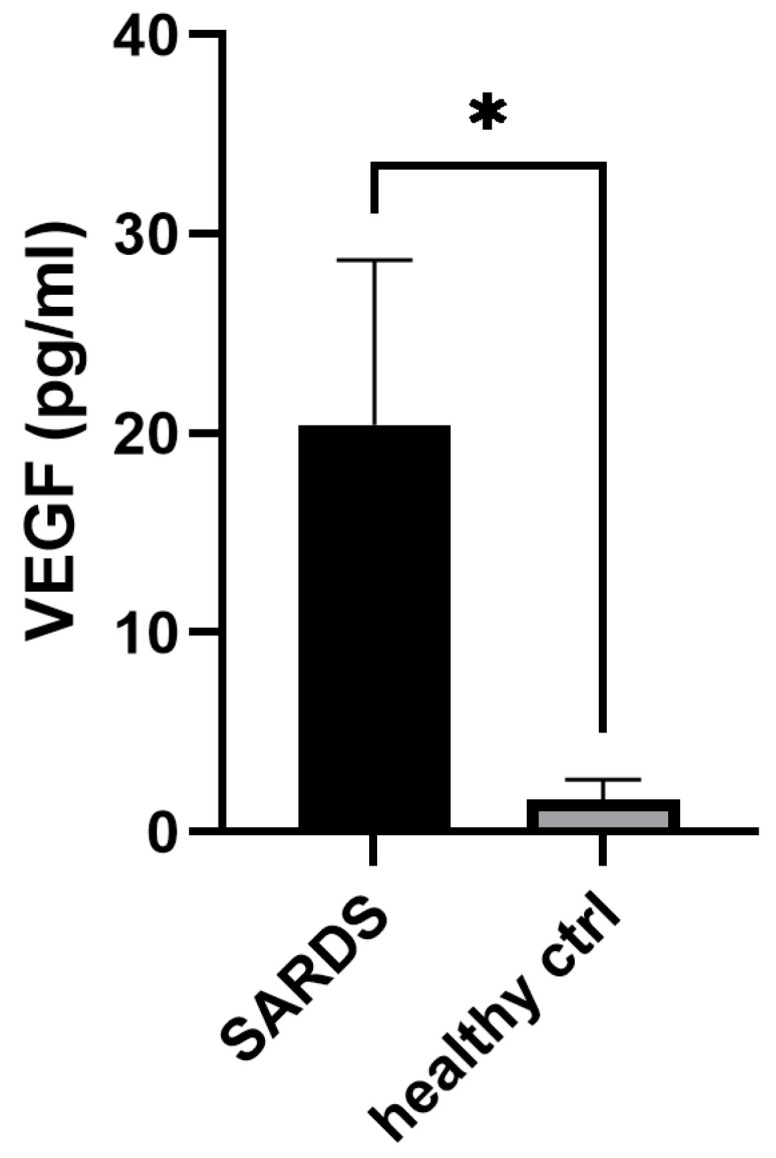
VEGF serum analysis in SARDS patients. VEGF serum analysis showed significantly increased VEGF levels in canine SARDS sera, when compared to healthy controls. * *p* < 0.05.

Evaluation of the serum levels of VEGF was pursued in canine SARDS and healthy canine sera samples with athegoal of determining whether VEGF serum levels are increased in SARDS patients. ELISA revealed elevated VEGF serum levels in SARDS patients (20.43 ± 8.2 pg/mL, mean + SEM, n = 59), which was statistically significantly higher (*p* = 0.02, Unpaired *t*-test, Figure 11) when compared to healthy control serum levels (1.6 ± 1. pg/mL, n = 19). ELISA revealed elevated VEGF serum levels in CAR patients (20.54 ± 15.6 pg/mL, mean + SEM, n = 5); however, the difference was not statistically significant when compared to healthy control serum levels (*p* = 0.21, Unpaired *t*-test,).

### 3.8. Identification of Potential Similarities Between SARDS and Human Retinal Diseases Based on the Microarray/Transcriptome Data and Clinical Symptoms Using the LLM Model

We have employed an LLM (Grok-4.20 Beta) to perform analysis of the raw SARDS microarray data with a goal of identifying similarities in transcriptome between SARDS and other human retinal diseases from previously published and publicly available microarray, transcriptome and proteome datasets from human retinal diseases. LLM analysis identified low similarity to retinitis pigmentosa (RP), medium similarity to age-related macular degeneration (AMD), and diabetic retinopathy (DR), and high similarity to autoimmune retinopathies (AIR) and cancer associated retinopathy (CAR) in humans (Table 3).

Furthermore, we have employed an LLM (Grok-4.20 Beta) to perform analysis of systemic and ophthalmic clinical symptoms for SARDS and retinal diseases in humans with a goal of determining whether analysis of clinical symptoms would provide additional information about the possible immune-mediated etiology of disease as suggested by DAVID and MetCore analysis of retinal transcriptome, and LLM microarray comparative analysis. Analysis of clinical symptoms provided the highest similarity to AIR/CAR human retinal diseases (Table 4).

### 3.9. Identification of Potential Similarities and Differences Between SARDS and CAR Based on the Microarray Data Analysis with Comparative Aspect to Human Autoimmune Retinopathies

We have employed an LLM (Grok-4.20 Beta) to perform analysis of the raw SARDS and CAR microarray data with a goal of identifying similarities and differences in transcriptome between SARDS and CAR canine retinal tissue. LLM analysis confirmed similarities (immune system activation, complement activation, photoreceptor death) and differences (cancer disease specific gene expression in CAR but not SARDS) between SARDS and CAR (Table 5). Observed LLM analytics data showed a high degree of similarities to data generated by DAVID and MetaCore analyses of retinal transcriptome (Table 5; Figure 5 and Figure 6).

### 3.10. ProetinBERT Deep Learning Model (DLMs) for Identification of Potential Drug Targets

We have employed ProteinBERT DLMs analysis of our GO and KEGG enrichment data with a goal of identifying therapeutic targets and possible candidate drug treatments. ProteinBERT DLMs were trained on three large drug-molecular interaction databases (DrugBank, BioSnap, and Therapeutic Target Database—TTD). ProteinBERT DLM analysis identified several classes of immunosuppressive and immunomodulatory drugs, which could be potentially utilized for treatment of SARDS and CAR (Table 6).

### 3.11. LLM Identification of Potential Drug Targets

We have employed an LLMs (Grok-4, ChatGPT4o) analysis of our GO and KEGG enrichment data with a goal of identifying therapeutic targets and possible candidate drug treatments. LLM analysis identified several classes of immunosuppressive and immunomodulatory drugs, which could be potentially utilized for treatment of SARDS and CAR, showing a significant overlap with DLM generated data (Table 7).

## 4. Discussion

This is the first study to provide a comprehensive transcriptomic comparison of Sudden Acquired Retinal Degeneration Syndrome (SARDS) and Cancer-Associated Retinopathy (CAR) in dogs, revealing shared immune-mediated molecular signatures as well as distinct disease-specific features. The combination of GO molecular function enrichment, KEGG pathway enrichment analysis, and transcription factor network mapping revealed a dominant activation of immune-related signaling in both conditions, with concurrent suppression of phototransduction and cardiomyocyte-related pathways. The transcription factor (TF) analysis supports an immune-driven pathogenesis for SARDS and CAR, with CREB1, SP1, PU.1, c-Jun, c-Myc, and NF-κB emerging as central TF regulators. These transcription factors orchestrate leukocyte chemotaxis, TNF signaling, and macrophage/dendritic cell activation, reinforcing the concept of an inflammatory microenvironment driving photoreceptor loss as previously demonstrated in human CAR and npAIR patients [1,3,5,23,24,25]. The identification of ESR1 and androgen receptor networks suggests that sex hormone signaling may also modulate retinal immune responses, a factor warranting further investigation, especially in the light of previously reported data showing elevation of serum sex hormones in SARDS patients [25,26]. The GO enrichment analysis highlighted overrepresentation of cysteine-type endopeptidase activity, peptidase activity, chemokine receptor binding, and MHC class II receptor activity. These findings point toward active proteolysis, likely related to apoptosis and antigen presentation, confirming previous study results demonstrating apoptosis of photoreceptors in SARDS retinas [27]. Upregulation of protein tyrosine kinase and oxidoreductase transcripts further supports the presence of altered intracellular signaling and oxidative stress, which are common features observed in many retinal neurodegenerative and inflammatory diseases [28]. The KEGG analysis provided a more disease-oriented view, revealing that the most significantly upregulated pathways (cell adhesion molecules, graft-versus-host disease, allograft rejection, and various autoimmune conditions) are all closely tied to T-cell activation and immune synapse formation. This aligns with prior evidence implicating cellular immunity in SARDS and CAR in dogs and humans [8,9,17,29,30]. In contrast, downregulated pathways are predominantly associated with cardiac muscle contraction, hypertrophic cardiomyopathy, and MAPK signaling, along with visual perception, likely corresponding to the photoreceptor loss as previously documented in canine SARDS and CAR patients [9,17,27,31]. The suppression of cardiac-related gene networks may reflect changes pertinent to the photoreceptor loss, since many of identified genes are also responsible for cytoskeleton protein synthesis in photoreceptors. However, these changes could also potentially reflect vascular remodeling as previously reported in npAIR patients, SARDS and canine CAR patients, or the presence of vascular inflammation sporadically reported in CAR patients [9,17,32,33]. When comparing the two conditions, CAR demonstrated higher expression of chemokine receptors CCR5 and CXCR4, consistent with paraneoplastic immune modulation. In contrast, SARDS exhibited stronger enrichment for complement and coagulation pathways (C5AR1), suggesting a more prominent role for innate immunity and vascular inflammation. A recent study of SARDS patients showed increased coagulability, further supporting the notion of a possible inflammatory vascular component along with platelet signaling abnormalities [34].

The elevated serum levels of vascular endothelial growth factor (VEGF) observed in SARDS patients, coupled with the pronounced retinal complement activation as evidenced by increased immunohistochemical expression of C1q and C4 components, provide compelling support for the immune-mediated etiology of the disease. VEGF, traditionally recognized for its role in angiogenesis, also exerts pro-inflammatory effects by enhancing vascular permeability and facilitating leukocyte extravasation, which may exacerbate immune cell infiltration into the retinal tissue, as corroborated by the presence of perivascular T- and B-cells in SARDS retinas [35]. This vascular-inflammatory axis aligns with the enriched KEGG pathways for complement and coagulation in SARDS, suggesting that innate immune activation via the complement cascade amplifies immune-mediated responses, potentially leading to epitope spreading and sustained photoreceptor damage. These findings not only align with an immune-mediated pathogenesis shared with CAR but also potentially highlight SARDS-specific vascular comorbidities, underscoring the potential for complement inhibitors as a potential treatment to mitigate immune-mediated retinal damage. Furthermore, elevated levels of VEGF are commonly observed in autoimmune diseases, where it plays a dual role in promoting angiogenesis and exacerbating inflammation, thereby contributing to disease progression and tissue damage [35,36]. In rheumatoid arthritis (RA), for instance, VEGF acts as a proinflammatory mediator that stimulates synovial angiogenesis, facilitating immune cell infiltration and joint destruction, with serum levels correlating strongly with disease activity and severity [35]. Similarly, in inflammatory bowel disease (IBD), increased VEGF expression links angiogenesis to chronic inflammation in the intestinal mucosa, promoting vascular permeability and leukocyte recruitment, which aggravates mucosal injury and ulceration [37]. This pattern extends to other conditions such as systemic lupus erythematosus (SLE) and multiple sclerosis (MS), where VEGF elevation correlates with active disease phases and may serve as a biomarker for monitoring therapeutic responses, highlighting its broader implications in autoimmune pathogenesis [35,36]. Considering that perivascular retinal lesions are frequently present in SARDS patients, it is possible that abnormal VEGF serum levels potentially contribute to vascular microdamage and increase vascular permeability for immune cells, diminishing the immune privilege provided by blood–retinal barrier [9].

To our knowledge, this is the very first study in the field of veterinary ophthalmology in which DLM and LLMs were used as exploratory methods to identify several hypothetical therapeutic avenues, including T-cell co-stimulation blockade (abatacept), complement inhibition (eculizumab, avacopan), leukocyte adhesion blockade (natalizumab), calcineurin inhibition (cyclosporine A, tacrolimus), and broad-spectrum immunomodulation (IVIg, corticosteroids). These findings align to some extent with previously published interventional data in humans and dogs and may offer a rational starting point for translational research and drug repurposing strategies [8,17,38,39,40,41].

Limitations of the study include the use of small-sample previously published microarray datasets. The DLM- and LLM-based drug predictions are exploratory/hypothesis-generating and will require experimental validation in future clinical treatment studies.

## 5. Conclusions

Overall, the transcriptomic landscape of SARDS and CAR reflects a convergence on immune-mediated mechanisms of retinal injury, with disease-specific differences in secondary inflammatory and vascular pathways. These data not only reinforce the autoimmune basis of both conditions but also provide a potential framework for more effective medical treatments for human and veterinary autoimmune retinopathies. Future work should focus on validating these pathway signatures in larger cohorts and assessing the in vivo effects of targeted immunotherapies.

## Figures and Tables

**Table 1:** KEGG pathway enrichment—shared upregulated gene sets from SARDS and CAR retinas.

Pathway	KEGG_ ID	Gene_ %	*p*-Value	Fold_ Enrichment	Benjamini	FDR	Genes
Cell adhesion molecules (CAMs)	cfa04514	13.95	0.000191	10.22	0.00197	0.1636	LOC607448, ALCAM, CD86, DLA-79, SELL, LOC480788
Graft-versus-host disease	cfa05332	6.98	0.005453	25.55	0.03333	4.5885	CD86, DLA-79, LOC480788
Allograft rejection	cfa05330	6.98	0.007383	21.9	0.03756	6.168	CD86, DLA-79, LOC480788
Autoimmune thyroid disease	cfa05320	6.98	0.011393	17.52	0.04948	9.3747	CD86, DLA-79, LOC480788
Type I diabetes mellitus	cfa04940	6.98	0.012029	17.03	0.04581	9.8743	CD86, DLA-79, LOC480788
Viral myocarditis	cfa05416	6.98	0.029655	10.57	0.0891	22.788	CD86, DLA-79, LOC480788

List of significantly enriched KEGG pathways among upregulated genes, including pathway name, KEGG ID, gene count, fold enrichment, and associated genes is shown. KEGG pathway enrichment showed predominant functional patterns associated with organ transplant rejection reaction and autoimmune diseases.

**Table 2:** KEGG pathway enrichment—shared downregulated gene sets from SARDS and CAR retinas.

Pathway	*p*-Value	Benjamini	Genes
Hypertrophic cardiomyopathy (HCM)	6.9 × 10^−6^	2.2 × 10^−4^	PRKAB1, CACNB2, TNNI3
Cardiac muscle contraction	1.5 × 10^−3^	2.4 × 10^−2^	CACNA1F, TNNI3
Visual perception	1.3 × 10^−4^	1.2 × 10^−2^	PDE6A, RP1, CNGA1

List of significantly enriched KEGG pathways among downregulated genes, revealed suppression of cardiac function and phototransduction pathways.

**Table 3:** LLM-predicted similarity between SARDS and human retinal diseases.

Human Retinal Disease	Key DEGs from Microarray/Transcriptomic Data (Up/Down)	Similarities to SARDS Microarray Data	Differences from SARDS Microarray Data	Level of Similarity (High/Medium/Low)
**Age-Related Macular Degeneration (AMD)**	**Macular AMD RPE/Choroid (Top examples)**: Up: FNDC1, LUM (ECM), LRRC15, PTPRZ1, CXCL14 (chemokine), TAF15, NGFR, IBSP, TNC (ECM), CHIT1; Down: DCAF6, SMOC1 (ECM), THPO, OST4, GAS1. Pathways: Neuroactive ligand-receptor interaction, ECM-receptor interaction (collagen genes like COL6A3, COL9A3 up).	Complement/ECM involvement (e.g., COL6A3 up in some AMD studies akin to SARDS complement C3 up); Oxidative stress/inflammatory genes (CXCL14 similar to SARDS immune activation).	Lacks strong immunoglobulin upregulation; Focus on ECM remodeling (LUM, TNC up) vs. SARDS immune/antibody genes; Crystallins not prominently up; More RPE-specific changes vs. SARDS photoreceptor/immune.	Medium (Shared complement and stress pathways, but less autoimmune focus).
**Retinitis Pigmentosa (RP, e.g., EYS-RP)**	Down: NRL (rod differentiation), CRYGD (crystallin), F2R (coagulation), LZTS1, SST, TNNC1, FOXS1, ANO2, HES6, NLRP12, KRT18; Also, phototransduction: OPN1SW, RCVRN, GNAT1, GNAT2, SAG, RHO down. Retina-related: CNGA1, COL11A1, SOX11, AIPL1 down; Apoptosis/ER stress: GJB2 down.	Some crystallin downregulation (CRYGD down vs. SARDS crystallins up, but both involve crystallins); Apoptosis/ER stress genes altered (similar to SARDS potential degeneration).	Predominantly downregulation of photoreceptor genes (RHO, NRL) vs. SARDS upregulation of immune/Ig; No strong complement or MHC I up; Genetic/degenerative focus vs. SARDS acquired/immune.	Low (Degenerative but opposite regulation in key visual genes; minimal immune overlap).
**Diabetic Retinopathy (DR)**	Key genes: Up: COL6A3 (collagen/ECM), IGFBP2 (insulin-like); Others: IGHG4 (Ig gamma), KLHDC7A, RPL26P30, MYL6P4 (regulation mixed, but COL6A3/IGFBP2 significantly up). Pathways: Circadian rhythm disruption, immune/inflammatory.	Some Ig involvement (IGHG4 similar to SARDS Ig chains up); ECM/collagen (COL6A3 up akin to SARDS complement/ECM links); Inflammatory/immune scores.	Vascular/angiogenesis focus (not in SARDS); Circadian genes (not prominent in SARDS); Less crystallin or MHC I emphasis; Metabolic/diabetic context vs. SARDS autoimmune.	Medium (Shared Ig and ECM, but DR more vascular/metabolic).
**Autoimmune Retinopathy (AIR, including CAR)**	Limited transcriptomic data; Proteomic (vitreous): Up: Lysozyme C, zinc-alpha-2-glycoprotein, complement factor D, TGF-β–induced, beta-crystallin B2, alpha-crystallin A; Down: DIP2C, retbindin, amyloid beta precursor-like protein 2. Pathway: VEGF signaling. (No direct human microarray; dog AIR/CAR shows immune/antigen presentation up, Ig synthesis up, complement up, similar to SARDS.)	Crystallins up (beta B2, alpha A similar to SARDS CRYBA2, etc.); Complement up (factor D akin to SARDS C3); Inflammatory/TGF-β (immune activation); In dog models: Strong Ig, complement, MHC/antigen presentation up (matches SARDS immune profile).	Proteomic vs. transcriptomic; Rare disease, scarce human data; Potential VEGF (not in SARDS); Down retbindin (photoreceptor-related, similar to SARDS degeneration).	High (Shared crystallin, complement, immune upregulation; AIR autoimmune etiology aligns with SARDS).

LLM analysis of previously published molecular data from human retinal diseases revealed the highest similarity between SARDS and autoimmune retinopathies in humans. DEGs—differentially expressed genes.

**Table 4:** LLM-predicted similarity between SARDS and human retinal diseases based on the previously published clinical symptoms.

Human Retinal Disease	Key Features	Similarities to SARDS	Differences from SARDS	Level of Similarity (High/Medium/Low)
**Autoimmune Retinopathy (AIR, including npAIR)**	Immune-mediated; autoantibodies against retinal antigens (e.g., recoverin, enolase); sudden or progressive vision loss; normal fundus initially; flat ERG; no inflammation signs; treatable with immunosuppressants in some cases.	High: Sudden blindness, anti-retinal antibodies, immune gene upregulation (Igs, complement), normal-appearing retina, flat ERG; proposed autoimmune etiology in SARDS; some response to IVIg/immunosuppression.	SARDS lacks confirmed autoantibodies consistently; AIR can be progressive; SARDS has stronger systemic endocrine signs (e.g., polyphagia, polydipsia); AIR not always associated with cancer (npAIR similar to non-cancer SARDS).	High (most similar based on immune mechanisms and clinical presentation).
**Cancer-Associated Retinopathy (CAR)**	Paraneoplastic; autoantibodies (e.g., anti-recoverin); sudden vision loss; photoreceptor degeneration; linked to underlying malignancy; poor prognosis.	High: Paraneoplastic autoimmune attack on retina; similar molecular profiles (immune activation, apoptosis); some dogs with SARDS-like symptoms have underlying cancer (IMR-CAR); gene expression shows immune similarities.	SARDS not associated with cancer; CAR often has detectable tumors; SARDS may have more aggressive immune profile; better therapeutic response in some dog CAR cases vs. SARDS.	High (especially for cancer-linked dog cases; SARDS often non-paraneoplastic).
**Bardet-Biedl Syndrome (BBS)**	Genetic ciliopathy; progressive retinal dystrophy (rod-cone); obesity, polydipsia/polyuria, hyposmia, renal issues; early onset.	Medium: Phenotypic overlap (retinal degeneration, obesity, polyphagia/polydipsia/polyuria, hyposmia); hypothesis that SARDS is acquired ciliopathy.	BBS genetic/congenital, slow progression; SARDS acquired, sudden onset in adults; no renal/genital anomalies in SARDS; ciliopathy in SARDS is hypothetical.	Medium (phenotypic but not etiological match).
**Alström Syndrome (AS)**	Genetic ciliopathy; progressive retinopathy; obesity, diabetes, hearing loss; early onset.	Medium: Similar to BBS; retinal degeneration with endocrine/metabolic signs (polyphagia, polydipsia).	AS genetic, progressive; SARDS sudden, acquired; AS has cardiomyopathy/hearing loss not seen in SARDS.	Medium (phenotypic overlap, recent hypothesis).
**Central Serous Chorioretinopathy (CSCR)**	Fluid accumulation under retina; focal detachments; associated with stress/hypercortisolism; acute vision disturbance; often self-resolving.	Medium: Retinal detachments common; hypercoagulable state; hypercortisolism-like signs; painless vision issues.	CSCR not typically permanent blindness; self-resolves in many; no flat ERG; not degenerative; SARDS irreversible.	Medium (systemic/hemostatic similarities, but ocular pathology differs).
**Age-Related Macular Degeneration (AMD)**	Chronic; drusen, RPE atrophy (dry), neovascularization (wet); oxidative stress, complement dysregulation; gradual central vision loss.	Medium: Immune/complement activation, oxidative stress, lipid metabolism issues; microarray overlaps (complement, proteostasis).	AMD gradual, age-related; affects macula; vascular in wet form; SARDS sudden, total blindness, no drusen/neovascularization.	Medium (shared pathways, but onset/progression differ).
**Retinitis Pigmentosa (RP)**	Genetic; progressive rod-cone dystrophy; night blindness, tunnel vision; bone spicules on fundus; variable onset.	Low: Photoreceptor degeneration; some immune involvement in advanced stages.	RP progressive, inherited; fundus changes (pigmentation); no sudden total blindness; no systemic endocrine signs.	Low (degenerative but different pattern/onset).
**Diabetic Retinopathy (DR)**	Vascular; microaneurysms, edema, neovascularization; linked to diabetes; progressive.	Low: Retinal damage in metabolic disorder context.	DR vascular/proliferative; associated with known diabetes; not sudden degenerative; no flat ERG early.	Low (metabolic link but different mechanisms).
**Acute Zonal Occult Outer Retinopathy (AZOOR)**	Acute; zonal photoreceptor loss; possible autoimmune; vision field defects; normal fundus initially.	Medium: Sudden outer retinal dysfunction; possible immune etiology; ERG abnormalities.	AZOOR zonal, not total blindness; may improve; more focal scotomas; less systemic signs.	Medium (acute outer retinopathy aspect).

Analysis of clinical symptoms provided the highest similarity to AIR/CAR human retinal diseases.

**Table 5:** LLM-analyzed similarities and differences between SARDS and CAR canine retinas.

Aspect	SARDS (Dogs) Microarray Data	CAR (Dogs) Microarray Data	Similarities	Differences	Notes on Human AIR/CAR
**Overall Gene Expression Profile**	Upregulated: Strong immune activation (Igs, complement C3, MHC class I, A2M); stress response (crystallins); complement/coagulation (C3, vitronectin); visual cycle (RBP3). Downregulated: Photoreceptor/visual genes indirectly (e.g., no direct down, but degeneration implied); some metabolic (e.g., not prominent).	Upregulated: Immunoglobulin-related (e.g., LOC475754, LOC608238); immune responses (antigen presentation, leukocyte activation); apoptosis/inflammation (e.g., FOS, JUNB, IL-6); PI3K-Akt/MAPK (e.g., PLA2G4A, NR4A1); cancer-associated (e.g., CDC2, ABCG2); metabolic (e.g., TKTL1, SLC2A3). Downregulated: TGFβ pathway (e.g., BMP4); innate immune (e.g., S100A8); lipid metabolism (e.g., APOC3); photoreceptor function genes.	Both show strong immunoglobulin upregulation and immune-mediated profiles; apoptosis/inflammation signaling common; metabolic shifts.	SARDS has stronger proinflammatory/chemokine (e.g., CXCL10, IL-18) and antigen presentation (e.g., DLA-DQA1, CD74); CAR emphasizes cancer/PI3K-Akt pathways; SARDS more aggressive immune profile.	No published human AIR/CAR microarray/transcriptomic data found; human CAR is rare, with mostly proteomic (e.g., anti-recoverin antibodies) or clinical studies.
**Number of DEGs**	Up: Many (e.g., >50 listed, focus on Igs/complement/crystallins); Down: >100 (e.g., photoreceptor-related indirectly via degeneration).	Vs. Controls: 107 probesets (83 genes) up, 95 (76) down. Vs. SARDS: 254 probesets (199 genes) higher in CAR, 167 (136) higher in SARDS.	Quantitative DEG overlap in immune genes.	SARDS has broader immune cell markers (T/B/NK); CAR has more cancer/metabolic DEGs.	Human AIR/CAR proteomic shows crystallins/complement up (similar to dogs), but no transcriptomics for direct comparison.
**Key Upregulated Genes**	Igs (LOC475754, IGHAC, J chain); Complement C3 (LOC476728); Crystallins (CRYBA2, CRYBA1); MHC I (DLA-88); A2M (LOC477699); Visual (RBP3).	Igs (LOC475754, LOC608238); Immune/apoptosis (IL-6, FOS, NR4A1); Cancer (CDC2, VEGFA); Metabolic (ALDH1A1, ARG1). Higher vs. SARDS: Igs, IL-6, VEGFA, FOS.	Shared Igs (e.g., LOC475754); apoptosis/inflammation (IL-6/FOS in CAR akin to SARDS immune).	SARDS unique: Complement C3, crystallins, MHC I; CAR unique: Cancer genes (CDC2), VEGFA.	Human AIR/CAR: Autoantibodies (anti-recoverin, enolase); no DEG data, but inferred immune/apoptotic from pathology.
**Key Downregulated Genes**	Visual/photoreceptor (e.g., RHO indirectly; some metabolic like not listed prominently).	TGFβ (BMP4); Innate immune (S100A8); Lipid (APOC3); Photoreceptor function. Higher in SARDS (i.e., lower in CAR): CXCL10, CD48, A2M.	Shared downregulation of photoreceptor genes implying degeneration.	SARDS has less TGFβ down; CAR downregulates more innate/lipid genes.	Human AIR/CAR: Photoreceptor loss (similar degeneration); no specific down DEGs reported.
**Enriched Pathways**	Adaptive/innate immune (B cell activation, complement); Oxidative stress (crystallins); Antigen presentation (MHC I).	Immune (antigen presentation, immunoglobulin); Apoptosis/inflammation; PI3K-Akt/RAS/MAPK; Cancer signaling; Metabolic (e.g., glycolysis). No complement upregulation.	Immune/antigen presentation; apoptosis/inflammation.	SARDS: Complement/coagulation, proinflammatory chemokines; CAR: Cancer/metabolic, negative TGFβ regulation.	Human AIR/CAR: Paraneoplastic immune (autoantibodies trigger apoptosis); pathways inferred as immune/apoptotic, similar to dog CAR.

LLM-analysis identified overlaps and differences in SARDS and CAR gene expression based on the analysis of microarray data. Similarities have been observed to previously published data from human AIR/CAR studies.

**Table 6:** DLM-predicted therapeutic targets and candidate drugs for SARDS and CAR.

Target Function	Drug Name	Gene	Drug Rank	Model Raw Score	*p*-Value	Source Database
T-Cell Costimulation	Abatacept	CD86	6/29,993	0.09	0.0005	TTD
			3/5017	0.51	0.0008	Biosnap
Complement Activation	Avacopan	C5AR1	12/29,993	0.32	0.0004	TTD
Leukocyte Adhesion	Natalizumab	ICAM5	13/5017	0.05	0.0024	Biosnap
		ALCAM	6/5017	0.30	0.0010	Biosnap
		VCAM1	9/5017	0.11	0.0015	Biosnap
TCR Signaling	Cyclosporine A	PPP3R1	3/5017	0.27	0.0010	Biosnap
		PPIA	2/5017	0.40	0.0009	Biosnap
		PPIF	2/5017	0.95	0.0003	Biosnap
		CAMLG	1/5017	0.98	0.0001	Biosnap
	Tacrolimus	FBKP1A	13/5017	0.98	0.0001	Biosnap
Autoimmune thyroid/diabetes-like signaling	IVIg	FCGR1A	1/5017	0.69	0.0007	Biosnap
		FCGR2B	1/5017	0.77	0.0006	Biosnap
	Methylprednisolone	NR3C1	3/1482	0.19	0.0039	Drugbank

DLM-predicted therapeutic targets and candidate drugs for SARDS and CAR revealed several immunosuppressive drug categories as potential candidates for the treatment of SARDS and CAR. Drug rank refers to the drug’s placement among all possible drug candidates available for a target gene. For instance, in the Biosnap database a drug rank of 3 can be viewed as significant as it indicates that only two drugs, among 5017, had a higher score for the target gene.

**Table 7:** LLM-predicted therapeutic targets and candidate drugs for SARDS and CAR.

Shared Pathway (Mechanism)	Statistical Significance	Representative Therapies
T-cell costimulation (CD86, DLA-79)	−log10(*p*) = 2.5	Abatacept (CTLA4-Ig)
Complement activation (C5AR1, classical & alt. paths)	−log10(*p*) = 3.1	Eculizumab, Avacopan
Leukocyte adhesion (CAMs, SELL)	−log10(*p*) = 2.8	Natalizumab, anti-LFA-1
TCR signaling	−log10(*p*) = 2.3	Cyclosporine A, Tacrolimus
Autoimmune thyroid/diabetes-like signaling	−log10(*p*) = 1.9	IVIg, corticosteroids

Summary of potential therapeutic targets identified through large language model (LLM) analysis of GO and KEGG enrichment data revealed multiple potential pharmacological strategies for treatment of CAR and SARDS.

## Data Availability

All research data are available upon the request to the corresponding author, Sinisa Grozdanic.

## Abbreviations

The following abbreviations are used in this manuscript:

**Abbreviation**	**Definition**
ACVO	American College of Veterinary Ophthalmologists
ACVP	American College of Veterinary Pathologists
AIR	Autoimmune Retinopathy
ALCAM	Activated Leukocyte Cell Adhesion Molecule
CAMs	Cell Adhesion Molecules
CAR	Cancer-Associated Retinopathy
CCR5	C–C Chemokine Receptor 5
CD	Cluster of Differentiation
C5AR1	Complement Component 5a Receptor 1
cfa	Canis familiaris KEGG species prefix
CNGA1	Cyclic Nucleotide-Gated Channel Alpha 1
CREB1	cAMP Response Element-Binding Protein 1
CTLA4-Ig	Cytotoxic T-Lymphocyte-Associated Protein 4–Immunoglobulin fusion protein
CXCR4	C–X–C Chemokine Receptor 4
DAVID	Database for Annotation, Visualization, and Integrated Discovery
DKFZ	German Cancer Research Center *(Deutsches Krebsforschungszentrum)*
DLA-79	Dog Leukocyte Antigen, allele 79
DVM	Doctor of Veterinary Medicine
ESR1	Estrogen Receptor 1
FDR	False Discovery Rate
GMP	Good Manufacturing Practice
GO	Gene Ontology
Grok-4	Grok large language model, version 4
HCM	Hypertrophic Cardiomyopathy
IRF8	Interferon Regulatory Factor 8
IVIg	Intravenous Immunoglobulin
KEGG	Kyoto Encyclopedia of Genes and Genomes
LFA-1	Lymphocyte Function-Associated Antigen 1
LLM	Large Language Model
MAPK	Mitogen-Activated Protein Kinase
MHC	Major Histocompatibility Complex
NF-κB	Nuclear Factor kappa-light-chain-enhancer of Activated B cells
npAIR	Non-Paraneoplastic Autoimmune Retinopathy
PDE6A	Phosphodiesterase 6A
PRKAB1	Protein Kinase AMP-Activated Non-Catalytic Subunit Beta 1
RelA	NF-κB Subunit RelA
RNA	Ribonucleic Acid
RP1	Retinitis Pigmentosa 1
SARDS	Sudden Acquired Retinal Degeneration Syndrome
SELL	Selectin L
SP1	Specificity Protein 1
PU.1 (SPI1)	PU.1 Transcription Factor
STAT1	Signal Transducer and Activator of Transcription 1
